# Editorial: Approaches to Address Resistance, Drug Discovery, and Vaccine Development in *Mycobacterium tuberculosis*: Challenges and Opportunities

**DOI:** 10.3389/fmicb.2022.871464

**Published:** 2022-04-29

**Authors:** Sandeep Sharma, Divakar Sharma, Nitin Pal Kalia

**Affiliations:** ^1^Department of Medical Laboratory Sciences, Lovely Professional University, Phagwara, India; ^2^Maulana Azad Medical College, University of Delhi, New Delhi, India; ^3^Department of Pharmacology and Toxicology, National Institute of Pharmaceutical Education and Research, Hyderabad, India

**Keywords:** *Mycobacterium tuberculosis*, drug discovery, vaccine development, drug resistance, resistance markers

*Mycobacterium tuberculosis* (*Mtb*), a causative microorganism for tuberculosis (TB), is considered to be a leading cause of mortality and morbidity around the globe. According to WHO, both active and latent forms of TB are major global health concerns, and efforts are being made to combat the emergence of *Mtb* infection at all levels. Even though effective treatments for TB are available, the health concerns to an individual with HIV co-infection and development of drug-resistant TB are the greatest concerns for researchers. In this special issue by Frontiers, our focus was to collect emerging ideas on new development in the field of drug discovery and vaccine development. Furthermore, we emphasized the emergence of drug-resistant strains of *Mtb* and their early detection. The current Research Topic aimed to focus on drug discovery and vaccine development, and manuscripts published in this issue updated the existing scientific knowledge of the researchers working in the field of *Mtb*.

The emergence of multidrug-resistant (MDR) *Mtb* leads to a serious health threat and financial drain to global health agencies. Alarmingly, MDR-TB cases in countries like India, China, and South Africa are on the rise and were estimated to be two to three times higher than figures from the last few years. The MDR bacteria have developed a formidable line of defense against existing antimicrobials *via* mutations in the target site, drug efflux, alteration in the target sites, and inactivation of drugs by enzymes. There is a pressing need to discover and develop novel drug molecules targeting continuously changing physiological and molecular mechanisms attributed to drug resistance and for effective eradication of MDR strains of *Mtb*. Effective combinational therapies and drug repurposing are among the latest choices to treat and mange MDR-TB.

After HIV, the present COVID-19 pandemic is an important key player in the emergence of MDR-TB and the ineffectiveness of current TB regimes. This is all due to the surge in immune-compromised patients, which leads to a huge surge in the global TB burden and causes a large number of deaths. The existing TB therapies are ineffective due to their long treatment time and less potent drugs with more side effects leading to the emergence of multidrug-resistant strains, which result in complex TB treatment. Zhou et al. screened a library of 4,000 peptide molecules in *vitro* and found orbifloxacin which can be used alone and in combination with first-line anti-TB drugs to improve their efficacy. Additionally, an attempt has been made to explore the computational approach for better efficacy in an *in vivo* study (Zhou et al.).

The emergence of drug resistance in *Mtb* to almost all the anti-TB drugs which are in use under clinical settings is being investigated and this research on clinical isolates of *Mtb* from the Portuguese population has updated the knowledge in the scientific community about streptomycin resistance, its transmission, and the rate of the mutation using next-generation sequencing data (Rocha et al.). Whole-genome sequencing data will help in understanding the resistance mechanism of two main first-line anti-TB drugs used in high burden countries like India, China, and South Africa (Tamilzhalagan et al.). Radiographic imaging of patients with MDR-TB was used for the first time for analysis in the Liupanshui Hospital (from January 2018 to December 2020) (Song et al.).

Tuberculosis management has become difficult due to the emergence of MDR *Mtb*. Dihydrosphingosine analogs (UCI) have shown promising results in mice models against sensitive and resistant bacterial strains. Detailed research is needed to accept this molecule as a therapeutic option. The study was designed to evaluate alterations in gene expression of three genes (gltA1, pry, and rspO) within clones of sensitive (H37Rv) and resistant (CIBIN: UMF: 15:99) strains of *Mtb* upon exposure to UCI-05 and UCI-14. Results revealed that exposure to UCI-05 upregulated gltA1 (2-fold) and lprQ expression (20%) in H37Rv and CIBIN: UMF:15:99 clones, respectively. Also, it downregulated rspO expression (11.08%) in sensitive H3Rv clones (Peñuelas-Urquides et al.). Alternatively, UCI-14 increased gltA1 expression (26%) and lowered lprQ expression (8.08%) among H37Rv clones with no change in gene expression of CIBIN: UMF:15:99 clones.

*Mtb* reprograms metabolic pathways to endure stress conditions. Toxin-antitoxin chaperone (TAC) systems encoded within the *Mtb* genome are activated under stress. The system is composed of HigA1 antitoxin, HigB1 toxin, and SecB-like chaperone (prevents HA1 degradation). This research assessed the HigB1 toxin's role in pathogenesis and stress adjustment. There was no correlation between bacterial growth and HigB1 toxin regulation during stress as shown in *in vivo* studies that revealed that there was upregulation in the expression of HigB1 toxin in stress conditions induced by the presence of levofloxacin and nutrient starvation (Sharma et al.). Compared to parental strains, guinea pigs infected with the HigB1 deleted strain showed reduced tissue damage and bacterial loads within the spleen and lungs during acute as well as chronic stages of infection, signifying the role of HigB1 in pathogenesis and adaptation.

*Mtb* has (p)ppGpp and PolyP as stringent response factors which help in generating a stringent response, a survival strategy adopted by bacteria to survive stress conditions and contributes to antibiotic tolerance, virulence, and persistence. Recent anti-TB approaches are extensive and oppressive. The review highlighted the current state of understanding stringent response, its role in virulence, persistence, and antibiotic tolerance, and the discussion of two advanced approaches intended to target stringent response. Treatment approaches such as the use of small molecule inhibitors (PPK1 & PPK2 inhibitors, RelA inhibitors like acetylated relaxin analog and pyrazinoic acid) and immune response-strengthening DNA vaccines (Relmtb DNA vaccine) display the potential to target stringent response and shorten TB treatment (Danchik et al.).

Kaufman summarized 140 years of progress in TB vaccine development. Drug treatment is the most effective available option but resistance to these drugs has forced researchers to choose alternatives. The development and utilization of new vaccines appear more hopeful and the search for an effective TB vaccine was started in the 19th century reporting more failures over success. The BCG vaccine, developed 100 years ago, is still the solely applied TB vaccine globally with the shortcoming of being effective only in neonatal extra-pulmonary TB and not effective for pulmonary TB, particularly among adolescents and adults. The present aim is to develop vaccines intended to improve or replace BCG. Subunit vaccine M72:AS01E has completed a phase IIb clinical trial with 50% protection toward active TB. Two viable whole-cell vaccine nominees, MTBVAC (currently in phase IIa clinical trial) and VPM1002 (phase III clinical trials), are also under investigation. The treatment approach focused on the combination of improved drugs and diagnosis, and a vaccine is required to control TB by 2030 (Kaufmann; Sharma et al., 2020).

## Author Contributions

SS was involved in writing, editing, and finalizing the manuscript. NK and DS edited the manuscript. All authors contributed to the article and approved the submitted version.

## Conflict of Interest

The authors declare that the research was conducted in the absence of any commercial or financial relationships that could be construed as a potential conflict of interest.

## Publisher's Note

All claims expressed in this article are solely those of the authors and do not necessarily represent those of their affiliated organizations, or those of the publisher, the editors and the reviewers. Any product that may be evaluated in this article, or claim that may be made by its manufacturer, is not guaranteed or endorsed by the publisher.
